# Effects of perioperative exercise on cardiorespiratory endurance in children with congenital heart disease in plateau areas after surgical repair

**DOI:** 10.1038/s41598-023-45310-0

**Published:** 2023-10-23

**Authors:** Ruixue Qi, Shijie Liu, Hongjie Wang, Xingwei He, Wanjun Liu, Fen Huang, Yujie Zhao, Bin Yang, Shunlin Xu, Hesong Zeng

**Affiliations:** 1 Division of Cardiology, Department of Internal Medicine, Tongji Hospital, Tongji Medical College, Huazhong University of Science and Technology, No. 1095, Jiefang Avenue, Wuhan, Hubei China; 2 Hubei Provincial Engineering Research Centre of Vascular Interventional Therapy, Wuhan, China; 3 Department of Cardiology, Zhengzhou Cardiovascular Hospital, Henan Medical Key Laboratory of Arrhythmia, Zhengzhou, China; 4 Department of Cardiology, Peking University Third Hospital, NHC Key Laboratory of Cardiovascular Molecular Biology and Regulatory Peptides, Key Laboratory of Molecular Cardiovascular Science, Ministry of Education, 49 North Garden Road, Beijing, 100191 China

**Keywords:** Cardiology, Health care, Medical research

## Abstract

We aimed to explore the effects of perioperative exercise on cardiorespiratory endurance in children with congenital heart disease (CHD) in plateau areas after surgical repair. Fifty children with CHD in the plateau admitted to our hospital were randomly divided into the exercise and control groups. The exercise group received a perioperative exercise intervention beginning within 24 h postoperatively, while the control group received routine nursing and treatment alone. To assess the 6 min walk distance (6MWD) at baseline and at end of intervention, children participated in a 6-min walk test before cardiac repair and at 1 week after general ward transfer. A subset of children in the study underwent the cardiopulmonary exercise test pre-operatively. The 6MWD of children with CHD at baseline was positively correlated with the peak oxygen uptake pre-operatively. No significant difference was reported in the preoperative baseline data of both groups. The 6MWD of the exercise group was significantly higher than that of the control group. Early exercise therapy after cardiac repair could significantly improve the cardiorespiratory endurance and exercise capacity of children with CHD in plateau areas.

## Introduction

Congenital heart disease (CHD) is the most common congenital disease in newborns; it is caused by abnormal development of the heart and/or large blood vessels in the fetus and includes heart wall, valve, and vascular malformations^1^. Advancements in surgical repair, percutaneous intervention, and medical management have significantly improved outcomes and survival, with more than 90% of children with CHD surviving to adulthood^2^. However, physiologic cure may not result in normal exercise ability in patients; the low exercise ability and cardiorespiratory endurance of these patients are likely to persist for a long time, thereby complicating the long-term prognosis^3^. Studies have demonstrated that children with CHD have lower physical fitness levels than their healthy peers^4^. Additionally, with increasing age, long-term comorbidities and complications are prone to occur, thus significantly increasing the risk of acquired cardiovascular disease and affecting their quality of life^5^. The hypobaric hypoxic environment of the plateau areas (altitude ≥ 3000 m in western China) results in higher incidences of CHD, and the pathological changes are more pronounced with the same disease conditions^6,7^. A multicenter study of the cardiorespiratory fitness of children with CHD showed that there was a significant difference in the maximal oxygen uptake (VO_2_ max) in this group, and the mean annual decline in VO_2_ max with age was significantly higher than that of the control group with no CHD^8^.

An active lifestyle is critical to the long-term health of patients with CHD. The current treatment of such patients no longer aims solely at short-term survival, however, the aim is long-term physical health, development, and well-being^9^. Cardiac rehabilitation is a carefully planned program that combines supervised physical activity, nutritional teaching, weight management, and lifestyle modification to help people with heart disease live healthier lives^10^. The goals of cardiac rehabilitation are to decrease patient symptoms, optimize cardiovascular risk factors, help regain strength, improve exercise endurance, and prepare for return to work or activities of daily living. Additionally, the goals include preventing worsening of heart disease and life-threatening events to help patients live longer^9^. Moreover, it is cost-effective^11^ and is rated as a class I recommendation with an A level of evidence by international clinical practice guidelines^12,13^. All people, regardless of their risk of cardiovascular disease, benefit from adequate physical activity^14^.

The Cochrane study^15^ has shown that, especially for CHD, pediatric cardiac rehabilitation is in its early stages. Currently, pediatric cardiac rehabilitation lacks a substantial good-quality evidence base. The impact of physical activity on patients with CHD has not yet been clearly assessed, and there is a lack of in-hospital exercise intervention studies of children with CHD^15^. The perioperative period after cardiac repair is a critical period comprising the possible recovery of cardiorespiratory endurance and exercise capacity, perioperative rehabilitation has great potential for optimizing cardiorespiratory and physical fitness, and the impact of early rehabilitation therapy on overall motor recovery is an important topic worldwide^16^.

The cardiopulmonary exercise test (CPET) has been increasingly used to preoperatively evaluate various diseases and treatment effects and formulate rehabilitation prescriptions, as it can evaluate exercise physiology, reflect the multisystem functions of the body in real-time during exercise, and objectively evaluate the patients’ cardiovascular function^8^. However, there is minimal relevant research data regarding CHD in Chinese children. Furthermore, the information relevant to children with CHD in the plateau areas is likely non-existent.

In cases where CPET is not feasible, such as inpatient settings or immediate postoperative period, the 6‑min walk test (6-MWT) is an alternative measure of cardiorespiratory endurance that has also been applied as the basis of function-based prognostic assessment^17^. The 6MWD and CPET indices demonstrated similar utility as univariate predictors for all-cause hospitalization/mortality and all-cause mortality^18^. However, the relationship between these two measures in children with CHD in plateau areas is most likely unknown.

Therefore, this study used device-based objective assessments and interventions to explore the characteristics of growth, development, and cardiorespiratory endurance of children with CHD in plateau areas. It evaluated the effect of an early exercise intervention after cardiac repair on the exercise capacity and cardiorespiratory endurance of these children. Ultimately, our aims were achieved.

## Methods

### Patient information

The study protocol was approved by the ethics committee of Zhengzhou Cardiovascular Hospital, affiliated to Southern Medical University (2020-10-002-F02). Patients in plateau areas who were admitted to the Cardiovascular Surgery Department of Zhengzhou Cardiovascular Hospital] between January 2020 and July 2022 were included. Parents or guardians of all of the patients provided signed informed consent prior to the study. The procedures followed were in accordance with the ethical standards of the relevant institutional committee on human experimentation and with the 1975 Helsinki Declaration, as revised in 2000.

#### Inclusion criteria

The inclusion criteria were CHD diagnosed using cardiac color Doppler ultrasound that required surgical repair after evaluation, the ability to independently complete the 6-MWT and exercise training procedure, aged less than 18 years, and signed informed consent by the parent or guardian.

#### Exclusion criteria

The exclusion criteria were unstable vital signs, the inability to perform exercise due to muscle and joint problems, severe instability of the airways (patients with severe emphysema), bronchial non-specifically marked hypersensitivity, and severe gas exchange impairment (total or partial respiratory insufficiency).

#### Withdrawal criteria

Patients could withdraw from the study voluntarily or if they were unable to complete the study procedures due to serious postoperative complications.

### Collection of basic clinical data

After admission, age, sex, height, and weight of each enrolled patient were collected. Body mass index (BMI) was calculated, and the left ventricular ejection fraction (LVEF) was assessed using echocardiography.

### Exercise ability assessment

#### Preparation of the patient

(1) The patient should wear comfortable clothing as well as appropriate shoes for exercise. (2) Patients should use their usual walking aids during the test (cane, walker, etc.) (3) The patient’s usual medical regimen should be continued. (4) A light meal is acceptable before early morning or early afternoon tests. (5) Patients should not have exercised vigorously within 2 h of the test’s start.

#### Preparation of the test

(1) Make sure the ambient temperature was kept at 20–25 °C with proper ventilation. (2) Repeat testing was performed at approximately the same time each day by the same professionally trained technician who was unaware of the groupings. (3) A “warm-up” period before the test should not be performed. (4) The serial number, name, age, height, and sex were accurately registered. (5) The patient should sit at rest in a chair, located near the starting position, for at least 10 min before the test starts. During this time, contraindications were assessed, pulse and blood pressure were measured, and clothing and shoes were confirmed to be appropriate.

#### Performing the cardiopulmonary exercise test

An incremental, symptom-limited CPET was performed on a cycle ergometer (Jaeger, German model: MasterScreen CPX)) with continuous measurements of oxygen consumption, carbon dioxide production, minute ventilation, heart rate, 12-lead electrocardiography, and oxygen saturation measured by pulse oximetry. Average resting indices were measured for 30 s prior to initiation of exercise. Blood pressure was calculated at rest and then every 2 min. Gas exchange data were measured breath-by-breath during the four stages of exercise at a pedaling rate of 60 revolutions per minute (rpm): (1) 3 min of rest, (2) 3 min of unloaded exercise, (3) a maximum incremental ramp (15 or 25 Watt/min), and (4) recovery with 5 min of unloaded cycling. The Borg 6–20 Rating of Perceived Exertion (RPE) scale was used to assess the perceived exertion and dyspnea.

The main exercise parameters were obtained at peak exercise points, corresponding to the highest work rate achieved and maintained for 30 s, consist of (a) load at peak, (b) peak oxygen consumption (VO_2_ at peak) as ‘Gold standard’ assessment of cardiorespiratory fitness, (c) peak VO_2_/kg was calculated as the ratio of peak oxygen consumption and body weight (VO_2_/kg at peak), (d) peak oxygen pulse was calculated as the ratio of peak oxygen consumption and heart rate (VO_2_/HR at peak), reflecting stroke volume and peripheral oxygen extraction, (e) peak ventilation (VE at peak), and (f) the VE/VCO_2_ slope was calculated from exercise onset to peak exercise by linear regression. The test was terminated when the participants exhibited symptoms that restricted their ability to continue the exercise, developed malignant arrhythmias, or had a Borg 6–20 RPE score of 17–19.

#### Performing the 6-MWT

The 6-MWT was performed on flat ground in a hospital room. In accordance with the guidelines of the American Society of Thoracic Surgery, the total length was 30 meters (m). Marks were made at every 3 m, and there were signs at the turn-back point. Participants were instructed to walk with the following information:

(1) Objective of the test: to walk as far as possible for 6 min. Running or jogging is not allowed. (2) Rest periods during the test: as 6 min is a long time to walk, the patient will be exerting him/herself. If he/she gets out of breath or becomes exhausted, they are permitted to slow down, stop, and rest as necessary. He/she may lean against the wall while resting, but he/she must resume walking as soon as he/she is able. (3) Route to walk (with a practical example by the physician or other trained personnel): position the patient at the starting line and stand near the starting line during the test. The physician/technician cannot walk with the patient. Invite the patient to start walking as soon as he/she is ready.

### Exercise training procedure

All children with CHD were postoperatively admitted to the intensive care unit. Within 24 h of being transferred from the intensive care unit to the general ward, the exercise group received exercise therapy based on the routine nursing treatment of the control group. The bedside power bicycle 30-min intervention program, which included 5 min of warm-up, 20 min of exercise therapy, and 5 min of recovery, was added to their treatment. The target heart rate during exercise was 20–30% higher than the basal heart rate (once per day). Breath-holding was avoided during the intervention process, and effective diaphragmatic breathing was incorporated. Cardiovascular specialists and rehabilitation therapists monitored and recorded the exercise process to ensure the safety of the participants.

### Statistical analysis

The primary hypothesis of the current study was that children who received a perioperative exercise intervention beginning within 24 h postoperatively would show significantly higher 6MWD than those who received routine nursing and treatment. According to our pre-experiment, based on a two-sided t-test with an *α* level of 0.05, a statistical power of 0.80, and a drop-out rate of 5%, the estimated sample size was 28 patients.

All statistical analyses were performed using SPSS software version 22.0 (IBM Corp., Armonk, NY, USA). Enumeration data are expressed as the number of cases and rates (%), and comparisons between groups of enumeration data were performed using the *c*^2^ test or Fisher’s exact test of independence. Normally distributed data are expressed as mean ± standard deviation. The independent samples *t*-test was used to compare continuous variables with equal variance between groups, and the paired *t*-test was used for comparisons within groups. *P* < 0.05 was considered statistically significant. The 6MWD, age, height, weight, BMI, and LVEF were compared between groups using the t-test, and the disease diagnosis and sex were compared between groups using the *c*^2^ test or Fisher’s exact test of independence.

## Results

**Figure 1:**
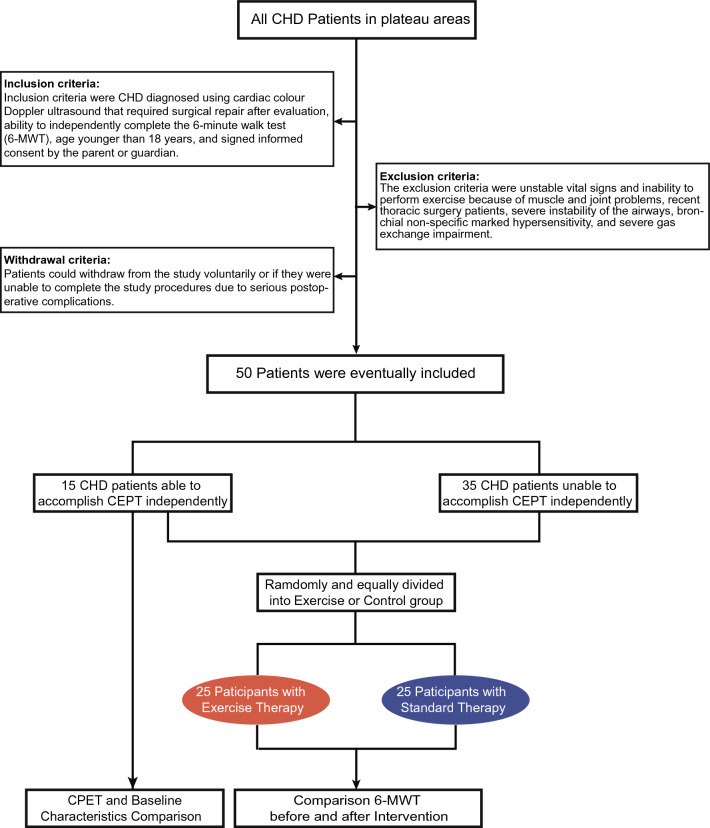
Flow charts of the procedures.

A total of 50 patients who met the inclusion criteria were included in this study, and all of them completed the requirements of the experimental protocol in a standardized manner, with no withdrawals. Among them, 15 were able to perform the CPET independently and complete it preoperatively. The 50 participants were randomly divided into the exercise and control groups, with 25 in each group. All participants participated in the 6-MWT to assess baseline and final mobilities and cardiorespiratory endurance before cardiac repair and after the intervention cycle (Fig. 1).

One patient developed paroxysmal atrial fibrillation postoperatively, and the exercise intervention was performed during sinus rhythm, and atrial fibrillation did not recur. The test process was safe, and no adverse cardiovascular events occurred.

### CPET for 15 children in two groups

Statistical testing revealed no significant differences in the demographic, LVEF, preoperative 6MWD and disease types. The CPET results exhibited no significant differences in load at peak, VO_2_ at peak, VO_2_/kg at peak, VO_2_/HR at peak, VE at peak, and VE/VCO_2_ slope between the exercise and control groups (*P* > 0.05) (Table 1).

**Table 1:** Demographic, LVEF, preoperative 6MWD, CPET and disease type of the total cohort and two groups.

	CHD	Exercise	Control	*t*/Z	*P*-value
Number	15	7	8	–	1.000
Female (%)	9 (60)	4 (57.14)	5 (62.50)	–	1.000
Age (years)	14.40 ± 1.06	14.29 ± 0.95	14.50 ± 1.20	− 0.386	0.706
Height (cm)	153.00 ± 9.36	154.14 ± 10.11	152.00 ± 9.23	0.427	0.677
Weight (kg)	42.07 ± 9.41	42.71 ± 10.09	41.50 ± 9.44	0.240	0.815
BMI (kg/m^2^)	17.76 ± 2.44	17.81 ± 2.72	17.71 ± 2.35	0.077	0.940
LVEF	64.80 ± 4.89	64.43 ± 3.95	65.13 ± 5.84	− 0.273	0.789
Preoperative 6MWD	414.73 ± 56.21	416.29 ± 64.03	413.38 ± 52.92	0.095	0.926
CPET					
Power at peak (w)	79.40 ± 25.53	84.00 ± 30.28	75.38 ± 21.86	0.625	0.545
VO_2_ at peak (mL/min)	1064.00 ± 311.40	1120.71 ± 400.33	1014.38 ± 224.80	0.622	0.549
VO_2_ at peak/kg (mL/min/kg)	25.28 ± 5.21	25.96 ± 6.87	24.69 ± 3.60	0.439	0.671
VO_2_/HR at peak (mL)	8.01 ± 2.09	8.20 ± 2.49	7.85 ± 1.84	0.306	0.765
VE at peak (L/min)	47.80 ± 13.68	50.71 ± 18.49	45.25 ± 8.07	0.724	0.490
VE/VCO_2_ slope	33.57 ± 2.82	32.31 ± 2.01	34.68 ± 3.08	− 1.778	0.100
Disease type				1.384	0.709
ASD	3	1	2	–	–
PDA	9	4	5	–	–
VSD	2	1	1	–	–
BAV	1	1	0	–	–

Data are expressed as the mean ± standard deviation or number (%).

CHD, congenital heart disease; BMI, body mass index; LVEF, left ventricular ejection fraction; 6MWD, 6 min walk distance; VO_2_, oxygen consumption; HR, heart rate; VE, ventilation; VCO_2_, carbon dioxide production; ASD, atrial septal defect; PDA, patent ductus arteriosus; VSD, ventricle septal defect; BAV, bicuspid aortic valve.

### The relationship of VO_2_ at peak and 6MWD pre-operatively

**Figure 2:**
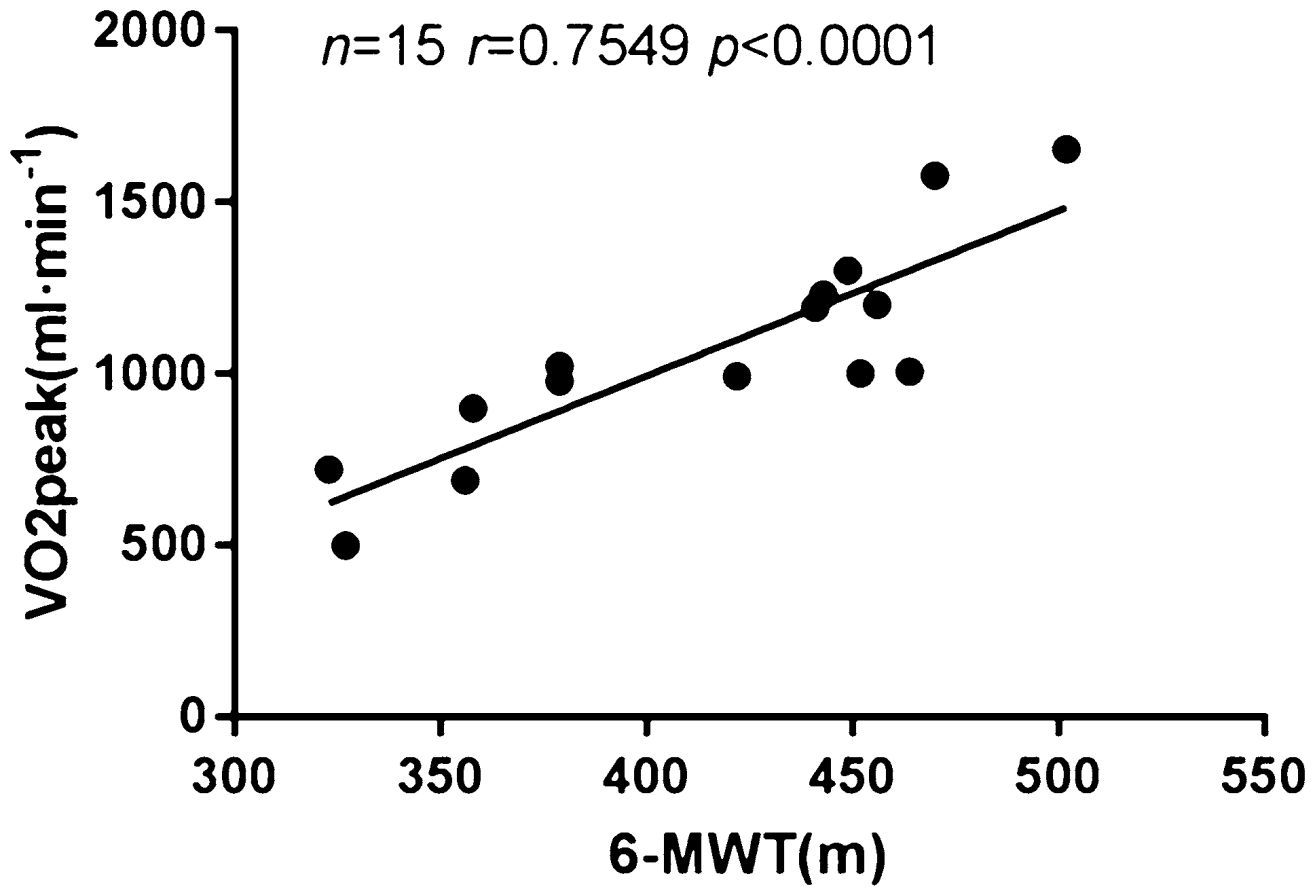
Correlation analysis of VO2peak and 6-MWT pre-operatively.

Preoperative VO_2_ at peak and preoperative 6MWD of the CHD were positively correlated (*r* = 0.7549; *P* < 0.0001) (Fig. 2).

### Exercise training in the two groups

Statistical testing revealed no significant differences in the preoperative general demographic data, LVEF, and disease type of the exercise and control groups (Table 2).

**Table 2:** Demographics, LVEF, and disease type of the total cohort and two groups.

	Total	Exercise	Control	t/χ	*P*
Number	50	25	25	–	–
Female (%)	35 (70)	18 (72)	17 (68)	4.0	0.261
Age (years)	10.60 ± 3.28	10.96 ± 3.09	10.24 ± 3.48	0.774	0.443
Height (cm)	134.96 ± 16.77	136.20 ± 15.52	133.72 ± 18.17	0.519	0.606
Weight (kg)	30.41 ± 10.81	30.94 ± 10.79	29.87 ± 11.02	0.347	0.730
BMI (kg/m^2^)	16.10 ± 2.10	16.16 ± 2.37	16.04 ± 1.85	0.193	0.848
LVEF (%)	65.68 ± 4.97	65.04 ± 4.73	66.32 ± 5.21	− 0.910	0.368
Disease type				2.000	0.157
ASD	11	6	5	–	–
PDA	30	14	16	–	–
VSD	6	3	3	–	–
BAV	3	2	1	–	–

BMI, body mass index; LVEF, left ventricular ejection fraction; ASD, atrial septal defect; PDA, patent ductus arteriosus; VSD, ventricle septal defect; BAV, bicuspid aortic valve.

**Figure 3:**
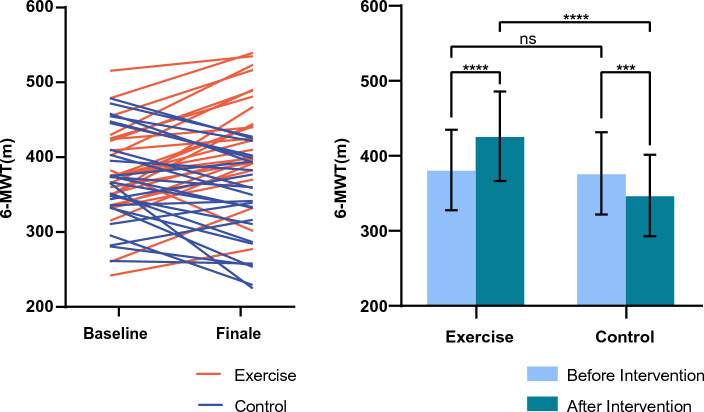
Baseline and final 6-MWT in the exercise and control groups.

There was no significant difference between the preoperative 6MWD of the exercise and control groups. After the intervention, the control group’s postoperative 6MWD was less than their preoperative 6MWD (376.56 ± 54.78 m vs. 347.12 ± 54.36 m; *P* < 0.001). Interesting, the intervention group had the opposite finding. The postoperative 6MWD of the exercise group was actually higher than their preoperative 6MWD (385.24.16 ± 55.46 m vs. 426.16 ± 59.75 m; *P* < 0.001) (Table 3 and Fig. 3).

**Table 3:** Comparison of the baseline and final 6MWD of the two groups.

	Baseline	Final	*t*	*p*
Exercise	385.24 ± 55.46	426.16 ± 59.75	6.363	< 0.0001
Control	376.56 ± 54.78	347.12 ± 54.36	4.248	0.0003
*t*	0.557	4.893		
*P*	0.580	< 0.0001

## Discussion

This study demonstrated that early exercise intervention (perioperative exercise) after cardiac repair significantly improved the cardiorespiratory endurance and exercise capacity of children with CHD in plateau areas. The test process was safe, and no adverse cardiovascular events occurred.

In our study, children with CHD in high-altitude areas showed lower performance in terms of VO_2_ at peak, VO_2_/kg at peak, and load at peak compared with previously reported measures in children with CHD in non-high altitude areas^8^. The results of this study suggest that environmental factors have a greater impact on the cardiorespiratory endurance of children with CHD. In plateau areas, especially high-altitude areas, changes occur in the oxygen transport systems, such as the heart and pulmonary blood vessels, to adapt to the hypobaric and hypoxic environments, maintain the best oxygen supply, and meet the oxygen consumption needs of tissues^6^. Therefore, it is of great importance to focus on the cardiorespiratory endurance of children in plateau areas. Strengthening the evidence base of CHD and cardiac rehabilitation after valve surgery and heart transplantation is a priority for future research^19^.

Furthermore, our study demonstrated that the 6MWD was significantly positively correlated with VO2 peak in children with CHD in plateau areas. CPET is generally regarded as the “gold standard” of aerobic assessment owing to its capacity to reliably discriminate differences along the continuum of low to high exercise performance. It can also identify and analyze the causes of exercise intolerance^20^. In contrast with CPET, the 6-MWT does not require complex equipment or technical expertise^21^. This low-complexity, safe test is suitable for people with serious diseases or those who cannot complete the CPET^22^. With the progressions in disease management, the discovery and treatment of CHD have advanced significantly, and most children with CHD are too young to independently complete the CPET^23^. The present study suggested that the CPET and 6MWD of children with CHD in plateau areas are correlated and that the 6-MWT can be used as an effective substitute and supplement for CPET in children with CHD in plateau areas.

There was no significant difference in the baseline data of the two groups of children with CHD in the plateau areas. After the exercise intervention, the 6MWD of the exercise group not only improved significantly compared with those of the control group, but also increased significantly compared with those of the control group before surgery, which showed a downward trend. Additionally, our findings did not reveal a significant difference in the effect of exercise training between different cardiac lesions. Possible explanations are that our sample size was small or the patients in our study had mild CHD phenotypes.

Regarding the 6-MWT, poor wound healing, and pain sensitivity of individual children in the exercise group affected the test results, but a significant overall improvement was observed. The possible underlying mechanisms for this are as follows: early postoperative exercise avoided the disuse-induced functional decline, strengthened the auxiliary function of the respiratory and muscle pumps, reduced cardiac work, increased myocardial perfusion, improved ventilation efficiency, and improved the body’s uptake and utilization of oxygen. These mechanisms have been verified by a study of patients with viral myocarditis and heart failure^24^. The cardiac repair corrected the abnormal blood circulation and improved hemodynamics and ventilation blood flow ratio, and exercise therapy promoted the overall recovery and improvement of the function and coordination of various body systems. The possible reasons for the decline in the control group were as follows: surgical trauma and the use of anesthesia and mechanical ventilation, including postoperative incision, limited lung function and physical activity. This resulted in decreased function, which offset the improvement in the exercise ability induced by surgical treatment. Furthermore, the combination of pulmonary hypertension and decreased cardiac contractility as well as the negative impact of cardiopulmonary bypass on the children’s postoperative exercise capacity could have occurred. A study of children with CHD with an average age of 268.4 ± 234.7 days reported that they are at risk for delayed gross muscle movements after cardiac surgery and that early postoperative exercise intervention can promote the recovery of motor function^15^; these results are consistent with our findings.

The survival of patients with CHD has improved dramatically in recent decades due to advancements in surgery and medical treatment. In the growing CHD population, acquired disease, especially acquired heart disease, has an increasingly important impact on the prognosis^8^. For patients with CHD, in addition to structural and functional abnormalities that make them more prone to atherosclerosis and adverse cardiovascular events^25^, persistently lower exercise capacity and muscle mass are important factors affecting the long-term prognosis. Numerous studies have demonstrated that lower physical activity levels are associated with higher rates of mortality and cardiovascular disease^26^ and that a low exercise capacity affects the health-related quality of life^27^.

Exercise capacity is an indicator of the CHD prognosis. Although there is clear evidence of the efficacy and safety of exercise training for pediatric patients, it is not performed as frequently as advisable^8^. No relevant studies have assessed the exercise capacity using an early exercise intervention for children with CHD^28^. There are only five randomized, controlled studies involving exercise therapy for children with CHD, however, all of them were performed in Europe and had small sample sizes, high loss to follow-up rates, and strong subjectivity. There is no data regarding an exercise intervention during hospitalization. Therefore, there is an urgent need for higher-quality studies with larger sample sizes^29^.

This study had some strengths. The exercise intervention during hospitalization could maximize the recovery of postoperative cardiorespiratory endurance as soon as possible. The short-term intervention and evaluation method based on the use of supervised equipment did not affect the discharge of children and greatly improved patient compliance. The quality of the intervention was guaranteed, and the experimental effect was evaluated objectively and quantitatively. The confidence of the children and families was enhanced, and the children’s psychosocial factors and self-cognitive functions were improved. The exercise patterns of the patients were stimulated as early as possible. A good experience with exercise could promote children’s exercise habits, which could continue into adulthood, and thus aid in disease prevention.

This study also had few limitations. The intervention and follow-up periods were short, and the impact of the intervention on the long-term growth and development of children could not be clearly defined. Long-term exercise programs after discharge were formulated for children in the exercise group, and regular follow-up contacts were established. However, it is still necessary to collect more relevant data regarding the cardiac rehabilitation of children with CHD in the non-plateau areas and to continue to deepen the research.

Overall, not only did we find that children who received perioperative exercise therapy had better 6-MWT, a test that we showed correlates to cardiorespiratory endurance, results than those who did not receive perioperative exercise therapy, but we found that their postoperative 6-MWT showed improvement over their preoperative 6-MWT. Perioperative exercise therapy after cardiac repair could restore and improve their cardiorespiratory endurance and activity. The intervention process was safe, and no adverse events occurred during this study.

## Data Availability

The datasets used and/or analyzed in this study are available from the corresponding author upon reasonable request.
